# Fog-Assisted Deep-Learning-Empowered Intrusion Detection System for RPL-Based Resource-Constrained Smart Industries

**DOI:** 10.3390/s22239416

**Published:** 2022-12-02

**Authors:** Danish Attique, Hao Wang, Ping Wang

**Affiliations:** 1 College of Computer Science and Technology, Chongqing University of Posts and Telecommunications, Chongqing 400065, China; 2 Department of Automation, Chongqing University of Posts and Telecommunications, Chongqing 400065, China

**Keywords:** Industrial Internet of Things (IIoT), fog computing, deep learning (DL), RPL, intrusion detection system (IDS)

## Abstract

The Internet of Things (IoT) is a prominent and advanced network communication technology that has familiarized the world with smart industries. The conveniently acquirable nature of IoT makes it susceptible to a diversified range of potential security threats. The literature has brought forth a plethora of solutions for ensuring secure communications in IoT-based smart industries. However, resource-constrained sectors still demand significant attention. We have proposed a fog-assisted deep learning (DL)-empowered intrusion detection system (IDS) for resource-constrained smart industries. The proposed Cuda–deep neural network gated recurrent unit (Cu-DNNGRU) framework was trained on the N-BaIoT dataset and was evaluated on judicious performance metrics, including accuracy, precision, recall, and F1-score. Additionally, the Cu-DNNGRU was empirically investigated alongside state-of-the-art classifiers, including Cu-LSTMDNN, Cu-BLSTM, and Cu-GRU. An extensive performance comparison was also undertaken among the proposed IDS and some outstanding solutions from the literature. The simulation results showed ample strength with respect to the validation of the proposed framework. The proposed Cu-DNNGRU achieved 99.39% accuracy, 99.09% precision, 98.89% recall, and an F1-score of 99.21%. In the performance comparison, the values were substantially higher than those of the benchmarked schemes, as well as competitive security solutions from the literature.

## 1. Introduction

The Internet of Things is a vigorously flourishing communication technology that introduces a new spectrum of smart communications [1]. It is extensively acknowledged for ensuring automated communication in a disseminated network of heterogeneous devices. A conventional IoT network incorporates various communicational nodes that are interlinked by tiny sensors [2]. Therefore, it makes up an integrated assortment of multitudinous devices that can mutually communicate regardless of any human interaction [3]. This phenomenal infrastructure of IoT endorses it as a substantial component of every smart communication environment. The miraculous performance of IoT can be witnessed in every sphere, such as in the educational sector, transportation sector, medical sector, agricultural sector, industrial sector, etc. [4,5]. In a traditional IoT network, the communication protocol plays a significant role, as it governs all of the communications among the participating nodes [6]. The Advanced Message Queuing Protocol (AMQP) [7], Message Queuing Telemetry Transport (MQTT) protocol [8], Long-Range Wide-Area Network (LoRAWAN) [9], and Sigfox [10] are some renowned communication protocols.

However, the Routing Protocol for Low-Power and Lossy Networks (RPL) is gaining significant attention. The Internet Engineering Task Force (IETF) designed an IPV6-based RPL protocol to expedite the routing mechanisms of resource-constrained networks [11]. This protocol operates at the physical standard of IEEE802.15.4 and is considered an optimal choice for ensuring reliable communication in low-power and lossy networks [12,13]. The application circle of RPL is expanding, and its applications can be observed in every IoT-based communication environment. The industrial sector is one of the highly privileged application areas of RPL, as the limited availability of resources and frequent communication breakages are crucial concerns in the industrial environment. RPL is a potential choice for coping with such emerging challenges in the industrial sector [14,15,16]. The expanding range of RPL applications in industrial sectors clearly shows its efficiency. However, these circumstances make RPL networks vulnerable to various potential risks [17]. Such challenging circumstances demand multiple security solutions in order to ensure durable and satisfactory communication in industrial networks [18]. Artificial intelligence (AI) [19], fog computing [20], software-defined networking (SDN) [21], machine learning (ML) [22], and deep learning (DL) [23,24,25] have been used to address this.

In the present era, combinations of deep-learning- and fog-computing-based schemes are considered fascinating solutions to overcome such challenges [26]. Fog computing provides a decentralized security approach by dividing the functional roles among various fog nodes. Hence, it prohibits the resource utilization of a particular node, making it an unusual approach to efficient resource management [27]. Secondly, it provides a two-layer-based surveillance architecture that is capable of conducting an investigation of malicious entities in a network [28]. Deep-learning-based approaches deliver an evolutionary mechanism for analyzing the traffic streams cascading over a network [29]. A DL-based system is first trained and tested on a dataset that contains existing impressions of an immense range of suspicious activities  [30]. There is a large catalog of training datasets, e.g., NSL-KDD [31], UNSW-NB15 [32], BOT-IoT [33], ADFA-LD [34], CICIDS2017 [35], and N-BaIoT [36], which can be employed to train the concerned systems. Further, such intrusion detection systems are used in virtual environments to investigate anonymous anomalies within networks [37]. These discernible advantages of both technologies motivated us to design a fog-assisted deep-learning-empowered intrusion detection system (IDS) for RPL-based resource-constrained smart industries.

### 1.1. Contribution

The significant contributions of this research work can be listed as follows:

- We have designed a fog-assisted deep-learning-empowered IDS, which is called the Cu-DNNGRU, to examine suspicious events in RPL-based resource-constrained smart industries.
- For the purpose of training, the proposed model was integrated with N-BaIoT, which significantly enhanced the detection capabilities of the designed framework.
- The established framework contains a combined sequence of the Cu-LSTMDNN, Cu-BLSTM, and Cu-GRU classifiers for comparison purposes, and they were trained and evaluated with the same dataset and performance metrics.
- The performance of the designed framework was also evaluated in comparison with some well-known benchmarked schemes.
- The authors also employed ten-fold cross-validation to show unbiased results.
- The simulation results support the validation of the proposed framework in terms of threat detection efficiency, accuracy, precision, resource consumption, and computational complexity.

### 1.2. Organization

This research study is organized systematically. Section 2 presents a delineation of related work. Section 3 describes the proposed security framework’s methodology, the elaboration of the datasets, and the simulation setup. Section 4 focuses on the results obtained after the performance evaluation of the proposed model, and the study is finally concluded in Section 5.

## 2. Related Work

RPL-based resource-constrained smart industries are attaining significant attention where notable efforts are being made towards its security. Here, we addressed some meaningful research studies surrounding this domain.

Authors [38] have proposed an IDS by including the appropriate elements of Light GBM to enhance the proposed system’s threat detection capabilities. The system is aligned with a customized dataset published by Oakridge Lab, which comes with a comprehensive variety of threat detection features. The same model is designed in [39], where a Convolutional Neural Network (CNN) based threat detection scheme is developed. The model is trained on two commonly known datasets, UNSW-NB15 and CICIDS2017. Simulations are carried out to evaluate the model’s validity, and researchers aim to assess this on the testbed. Another attempt is made in [40], where researchers have focused on the combined strength of some well-known classifiers such as the Long Short-Term Memory (LSTM) and the Gated Recurrent Unit (GRU). They have proposed a hybrid model that is trained on the N-BaIoT dataset and is capable of interrogating malicious events in resource-constrained environments. Likewise, a hybrid intrusion detection framework is proposed by using LSTM classifier [41]. UNSW-NB15 and NSLKDD datasets are acquired to train the system, and the system’s efficiency is evaluated in a pervasive simulation environment. Researchers have obtained the generic features of the CNN classifier to design an anomaly detection mechanism in the healthcare environment. The framework is trained on the CICIDS2017 dataset and analyzed using rational performance metrics. The system has potentially identified a range of emerging cyber threats in the smart healthcare environment [42]. Another model is designed by using a Deep Neural Network (DNN) based classifier and is potentially trained on UNSW-NB15 and NSLKDD datasets. The obtained result validates the importance of the proposed model [43]. Authors have used Text-CNN classifiers and the KDD99 dataset to propose a threat detection model for smart industries [44].

Single-Hidden Layer Feed-forward Neural Network (SLFN) is one of the best classifiers for responsive intrusion detection in industrial environments. It, with the LSTM classifier, brings additional strength to the system.

Researchers have adopted these two classifiers to design a multifunctional threat classification mechanism. Furthermore, the IoT-ID20 dataset is used for training purposes [45]. Another effective threat classification scheme is presented [46], where authors mainly target the less frequently occurring suspicious events in industrial networks. They have observed the real-life scenario for a sustainable period and have organized a customized dataset. The N-BaIoT data set is also integrated into sequence with their customized dataset. Their model is facilitated with Principal Component Analysis (PCA) and deep learning classifiers that offer additional support to instantly identify these attacking circumstances. Researchers in [47] provide an alternative deep learning-based detection mechanism. Multiclass classifiers accompany the BOT-IoT dataset for a highly accurate investigation of suspicious entities. The system has achieved remarkable accuracy in distinguishing between normal and abnormal traffic. For RLL-based smart communication industries, a deep learning-inspired malicious packet filtering mechanism is provided [48]. Researchers have used an embedded DNN classifier that controls the entire processing infrastructure. The proposed approach is capable of handling Denial of Services DoS and port scan attacks. Researchers have designed a multidimensional system consisting mainly of a forest PS classifier to investigate crucial security threats in resource-efficient smart industrial environments. They have used a CICIDS2017 dataset containing details of potentially harmful events. The designed model obtained impressive accuracy with high precision and an F1 score [49].

The authors present a deep learning-based IDS developed on a custom dataset. The proposed scheme utilizes Multilayer Perceptron (MLP), Decision Tree (DT), and LSTM classifiers to enable efficient intrusion detection. While performing simulations, the proposed framework has projected splendid performance on analytical performance metrics [50]. Researchers have used the Classification and Regression Tree (CART) classifier and CNN to present a dynamic security framework that ensures instant recognition of suspicious events causing security breaches. The scheme has been trained on the NSL-KDD and the KDD-99 dataset, achieving sustainable performance to safeguard resource-constrained smart industrial environments [48]. The related work is summarized in Table 1.

## 3. Methodology

### 3.1. Proposed System Architecturem

The authors designed an IDS composed of two charismatic technologies, deep learning and fog computing, where both technologies are assigned specific roles. Deep learning participates in intrusion detection activity, whereas fog computing provides an ideal infrastructure to implement that deep learning-based intrusion detection system. Fog computing also offers a systematic architecture in which different tasks are divided among various communication nodes according to their resource occupancy. RPL-based communication networks are just an application area for which we have proposed this IDS. This way, a comprehensive mechanism is formulated where both these technologies, i.e., deep learning and fog computing, rub shoulders together to perform intrusion detection in RPL-based resource-constrained smart industries.

**Figure 1:**
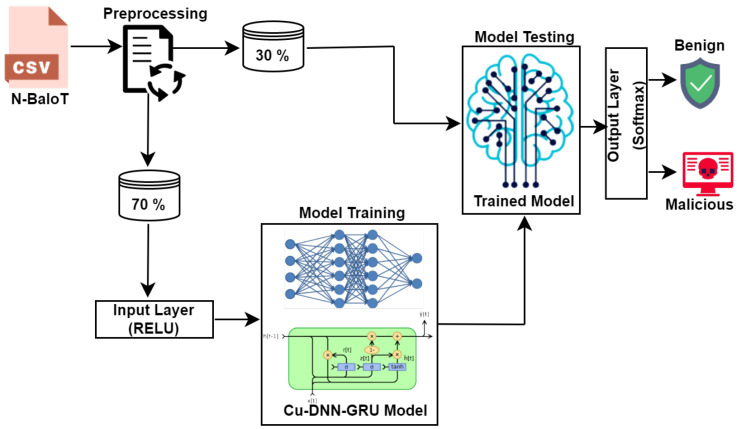
Work flow of proposed detection scheme.

In the proposed detection framework, DNN participates with four layers of neurons bearing 400, 300, 200, and 50 layers of neurons, whereas GRU contributes with two layers carrying 200 and 100 neurons, respectively. As shown in Figure 1, the active function RELU is employed for both classifiers DNN and GRU; however, the dynamic function softmax is integrated at the output layer. The scheme is occupied with Adam optimizer to acquire the desired performance objectives. For a classified analysis of the system’s performance, the designed Cu-DNNGRU is tested, with Cu-LSTMDNN having two layers of neurons, BLSTM, and GRU with four layers of neurons for each classifier. The proposed framework is evaluated on an analytical performance scale where simulations are carried out to 15 epochs with a batch size of 32. The comprehensive elaboration of the proposed intrusion detection framework is further enlisted in Table 2.

### 3.2. Algorithm Description

In the proposed IDS, the deep learning-based algorithm purely focuses on the intrusion detection activity and effectively interrogates anomalous events in RPL-based resource-constrained smart industries. The proposed Cu-DNNGRU is an amalgamation of two prestigious classifiers, Deep Neural Networks (DNN) and Gated Recurring Unit (GRU). A deep Neural Network (DNN) is an enhanced version of an Artificial Neural Network (ANN) with extra layers. The layers of neurons are organized in a sequence of multiple layers, where neurons receive the neuron activations from the previous layer as input and perform a simple computation. Each neuron receives a set of *x*-values numbers from 1 to *n* as an input and computes the predicted *y*-hat value. Vector *x* contains the values of the features in one of the *m* examples from the training set. In each iteration, the neuron calculates a weighted average of the values of the vector *x* based on its current weight vector *w* and adds bias. Finally, the result of this calculation is passed through a non-linear activation function.

(1) $$Z=Bias+W_{1}X_{1}+W_{2}X_{2}+W_{3}X_{3}+\cdots +W_{n}X_{n}$$

The Gated Recurrent Unit (GRU) is an advanced version of the Recurrent Neural Network (RNN) and is quite similar to the LSTM. GRU also utilizes gates to regulate the information flow. They choose which information should be sent to the output and are referred to as the two vectors. Its specialty is storing old data rather than getting rid of it since it is not essential to the forecast. GRU consists of an update gate $\left(U_{t}\right)$, current memory state $\left(h_{t}\right)$, and reset gate $\left(r_{t}\right)$.

(2) $$U_{t}=\left(W^{z}+X_{t}+U^{z}h_{t-1}+B_{z}\right)$$

where $X_{t}$ is the input multiplied by the weight $W_{z}$. Further, $h_{t-1}$ holds the information of the previous state multiplied by its weight $U_{z}$. For computing $r_{t}$, Equation (3) is used.

(3) $$r_{t}=\sigma \left(W^{\left(r\right)}+X_{t}+U^{\left(r\right)}h_{t-1}\right)$$

where σ represents the sigmoid function, $r_{t}$ is the reset gate, $W^{r}$ is the weight, $x_{t}$ is input, and so on. It then uses Equation (4) to store all the relevant information from the past, where ⊙ is class-wise multiplication, $h_{t}^{\prime }$ is the information from the previous stages and $h_{t-1}$ is the current memory content.

(4) $$h_{t}^{\prime }=\tanh\left(WX_{t}+r_{t}⊙\left(U\right)h_{t-1}\right)$$

Finally, Equation (5) is used where the network calculates the $h_{t}$.

(5) $$h_{t}=Z_{t}⊙h_{t-1}+\left(1-Z_{t}\right)⊙h_{t}^{\prime }$$

The complete workflow of the proposed detection scheme is depicted in Algorithm 1.
**Algorithm 1** Proposed hybrid detection framework.1:**Input**: Dataset = DTS2:**Output**: Benign→0, Attack1→1 and so on.3:Split the DTS in to $DTS_{TrainingData}$ and $DTS_{TestingData}$4:**for**each layer of DNNGRU**do**5:   ${DTS}_{Train}^{\prime }$ = Pre-proceessing of $DTS_{TrainingData}$6:   $DNNGRU_{Training}$ = Train the model using ${DTS}_{Train}^{\prime }$7:   $Z=Bias+W_{1}X_{1}+W_{2}X_{2}+W_{3}X_{3}+........+W_{n}X_{n}$8:   $U_{t}=\left(W^{z}+X_{t}+U^{z}h_{t-1}+B_{z}\right)$9:   $r_{t}=\sigma \left(W^{\left(r\right)}+X_{t}+U^{\left(r\right)}h_{t-1}\right)$10: $h_{t}^{\prime }=\tanh\left(WX_{t}+r_{t}⊙\left(U\right)h_{t-1}\right)$11: $h_{t}=Z_{t}⊙h_{t-1}+\left(1-Z_{t}\right)⊙h_{t}^{\prime }$12:**end for**13:$DTS_{Test}^{\prime }$ = Pre-processing of $DTS_{TestingData}$14:$DNNGRU_{Testing}$ = Test the model using $DTS_{Test}^{\prime }$15:**while** True **do**16: $Predict\;Attack\to DNNGRU_{TModel}\left(DTS_{Test}^{\prime }\right)$17: **if** the value predicted = 0 **then**18:  Return Benign19: **else**20:  Return attack type21: **end if**22:**end while**

### 3.3. Proposed Network Model

**Figure 2:**
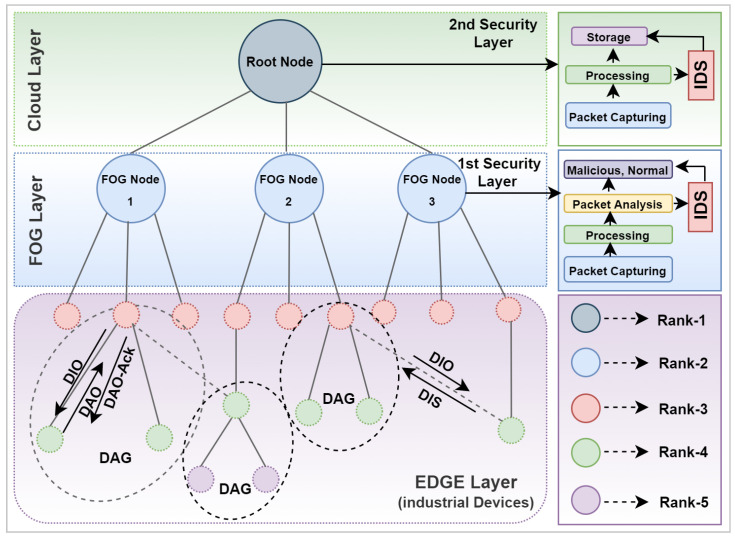
Proposed network model.

The proposed threat detection mechanism is massively privileged by fog computing in terms of operational architecture. In resource-constrained smart industries, the operational role needs to be assigned according to the resource occupancies of the concerned nodes. Hence, fog computing provides an impressive infrastructure where communication nodes are categorized into various layers that are indulged in the cloud layer, the fog layer, and the edge layer, respectively. Starting from the bottom, the edge layer comprises a scattered dimension of RPL nodes identically tied up in organic clusters. The second layer is the fog layer, which administers the functionalities of the edge layer and offers substantial durability to the system by assisting with optimal routing streams within the network. The fog layer is then in coordination with the cloud layer which supervises the functionalities of the fog layer and performs superior functionalities such as data storage, extinguishable administration, etc. That phenomenon squarely tends to yield highly productive management of system resources. The deep learning-based threat investigation approach aggregately works in coordination with fogging. The threat detection model is originally trained on a comprehensive dataset to make it conceived with various generic impressions of security threats. Henceforth, the framework is implemented on the fog and cloud layer. The fog layer persuasively flags all the suspicious events from the edge layer. However, the fog nodes also possess a probabilistic risk of being compromised. Such uncongenial circumstances may question overall security, reliability, and efficiency of the whole communication network. That phenomenon stimulates the need of a backup plan to cater these unexpected misshaping. Hence, we have introduced two layers of security that leverage an extended security ecosystem. This fog layer serves underneath the cloud layer, so the unaddressed security concerns are then consequentially dismantled by the cloud layer. The overall proposed network model can be witnessed in Figure 2.

### 3.4. Dataset Description

The dataset is a substantial element of every DL-based intrusion detection scheme [52]. Selecting an effective and proportionate dataset significantly strengthens the IDS. The dataset selection depends on where the IDS will be implemented [53]. An extended range of datasets is available. A vast variety of auxiliary datasets may coordinate with intrusion detection approaches such as ADFA-LD, NSL-KDD [54], BOT-IoT [55], etc. The proposed IDS is designed for RPL-based communication networks. So, the dataset must be closely relevant to this application area. Hence, the proposed detection scheme is trained on the N-BaIoT dataset, which is a suitable choice for intrusion detection in industrial environments. The N-BaIoT dataset contains comprehensive impressions of security threats frequently happening in RPL networks. The N-BaIoT dataset is comprehended with 94,914 attack instances, among which 61,400 are regular attacks. In comparison, the other cases are tied up with crucial security threat categories such as Mirai Scan, Mirai UDP, Mirai Ack, Gafgyt junk, Gafgyt combo, Gafgyt TCP, etc. The dataset details are further elaborated in Table 3.

### 3.5. Dataset Pre-Processing & Normalization

Pre-processing involves the standard mechanism to organize the data in a usable form, such as gratuitous spaces and eliminating non-value entries. The N-BaIoT dataset is pre-processed to achieve great utility through the sklearn pre-processing label encoder. The deep learning algorithm solely reckoned on numeric values; the sklearn label encoder has converted all non-numeric values to numeric entities. Dataset normalization converts all numeric columns to the same scale without changing the range of values. It is only necessary to normalize datasets with a wide range of values. Minimax Scalar is used to normalize the N-BaIoT dataset, which is generally scaled to a predetermined range between zero and one. The suggested model performs better and yields more valuable results with a normalized dataset.

### 3.6. Experimental Setup

The proposed scheme’s empirical performance test is conducted on an 8th-generation computer machine furnished with a 3.33 GHz processor, 16 GB RAM, and a Windows 10 operating system. The Graphical Processing Unit (GPU) used for simulations is Geforce-1060, equipped with Python as a programming language and Numpy, Tensorflow, Pandas, Keras, and Scikitlearn libraries. The experimental setup can also be overviewed in Table 4.

### 3.7. Simulations Parameters

The proposed DNNGRU framework has been evaluated on a comprehensive performance matrix including accuracy, precision, recall, ad f-Score as simulation parameters. The accuracy of a system is calculated by the accumulative summation of the True Positives (TP), the True Negatives (TN), the False Positives (FP), and the False Negatives (FN). The recall is considered an essential element in ascertaining the system’s performance. It denotes the average number of correct analyses released by an algorithm. In some cases, the term precision swapped places with recall because it affirms the accumulative projected by a framework.

## 4. Results and Discussions

**Figure 3:**
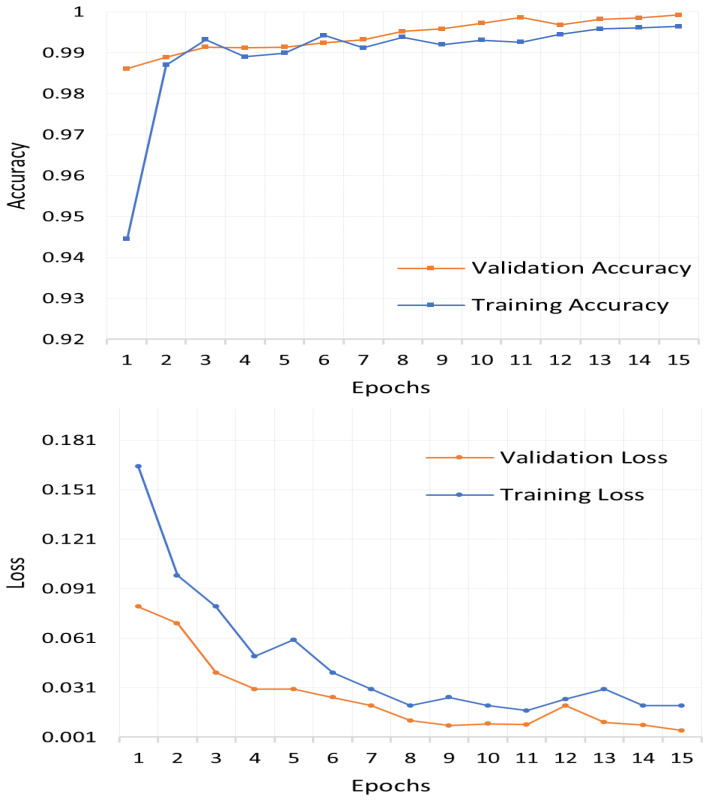
Validation accuracy vs. validation loss.

**Figure 4:**
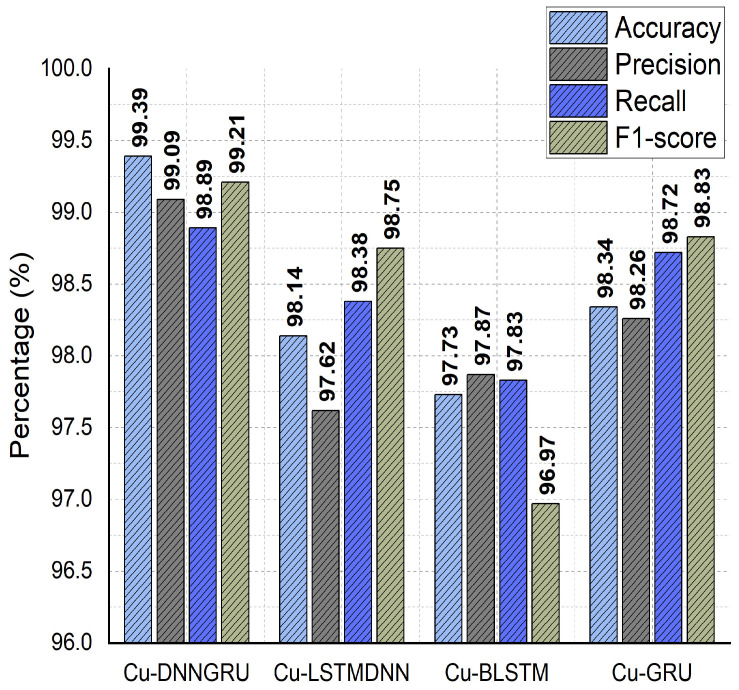
Accuracy, precision, recall, and F1-score analysis.

The performance of the proposed Cu-DNNGRU is evaluated concerning other competitive classifiers, i.e., Cu-LSTMDNN, Cu-BLSTM, and Cu-GRU, under a reasonable performance matrix equipped with accuracy precision, recall, and f-Score. DNN-GRU was able to effectively learn from the dataset, as evidenced by the results produced in terms of accuracy vs. loss, as shown in Figure 3. The validation results for the model were 0.025% validation loss and 99.39% validation accuracy. Moreover, on a comparative performance scale, the proposed Cu-DNNGRU projects a phenomenal performance by achieving an overall accuracy of 99.39%, 99.09% precision, 98.89% recall, and 99.21% F1-score as witnessed in Figure 4. We have further provided the class-wise detection rate of the proposed model against the other models in Table 5.

The proposed Cu-DNNGRU is evaluated on a ten-fold cross-validation under an investigative variety of performance parameters such as accuracy, precision, recall, and F1-score. It can be seen in Table 6 that Cu-DNNGRU has achieved remarkable performance in comparison with Cu-LSTMDNN, Cu-BLSTM, and Cu-GRU. Regarding the accuracy, the DNNGRU maintains the first-fold accuracy of 98.61%, 98.21% precision, 99.65% recall, and 98.99% F1-score. The progression continues in almost the same fashion until the 10th fold, where Cu-DNNGRU has a projected accuracy of 99.92%, 98.71% precision, 99.12% recall, and 99.81% F1-score.

**Figure 5:**
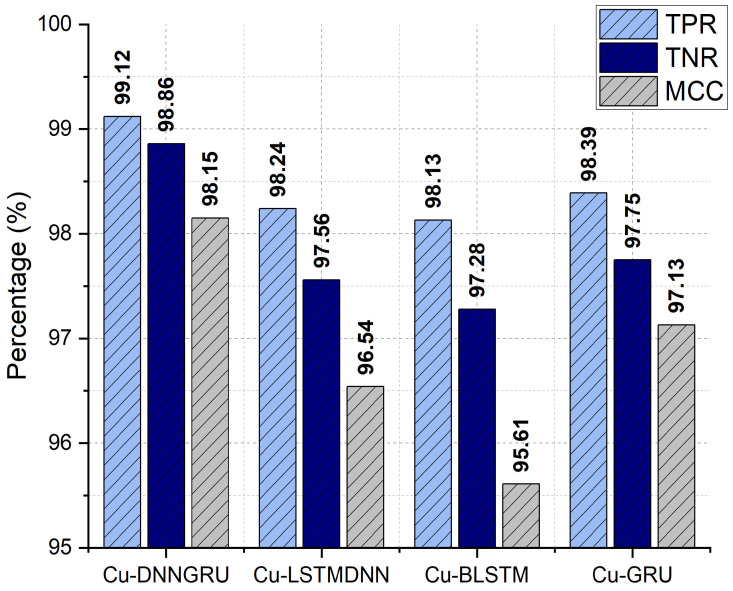
TPR, TNC, and MCC Analysis.

Accommodating a broader variety of assessment metrics, comprising the True Positive Rate (TPR), True Negative Rate (TNR), and Matthews Correlation Coefficient (MCC), the proposed Cu-DNNGRU is evaluated in comparison with Cu-LSTMDNN, Cu-BLSTM, and Cu-GRU. Figure 5 depicts that Cu-DNNGRU has shown a TPR of 99.12%, which is significantly exceptional compared to other competitive schemes. Moreover, in the case of TNR, Cu-DNNGRU again advertised an admirable performance with a TNR value of 98.86%. The relevant sequence exclusively goes on when the proposed CU-DNNGRU deliberates an exceptional MCC value of 98.15%.

**Figure 6:**
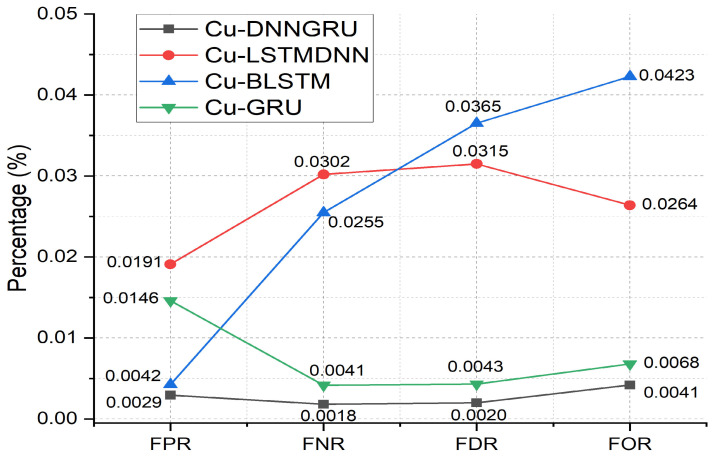
FPR, FNR, FDR, and FOR analysis.

We have further investigated the performance of the proposed Cu-DNNGRU on rational performance metrics, including False Positive Rate (FPR), False Negative Rate (FNR), False Detection Rate (FDR), and False Omission Rate (FOR). It can be seen in Figure 6 that the proposed Cu-DNNGRU has achieved the FPR of 0.00293%, and the number is considerably less as compared to other benchmarked technologies. The low value of FPR declares the superiority of the proposed framework. The next crucial performance parameter is FNR. Cu-DNNGRU projects an FNR of 0.00183%, which is less than the FNR that other benchmarked technologies achieve. On a comparison at FDR, Cu-DNNGRU exhibits substantial performance with a value of 0.00200%. Cu-DNNGRU again illustrates a dominance over other competitive technologies with a FOR discount of 0.00419%.

**Figure 7:**
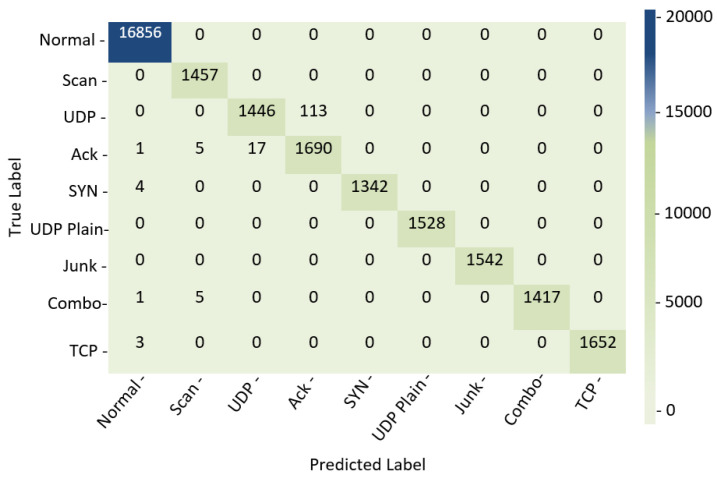
Confusion matrix analysis.

Furthermore, a confusion matrix summarizes the performance of a DL-based classification algorithm. Calculating a confusion matrix can provide a more accurate perspective of the categorization model’s accomplishments. The proposed Cu-DNNGRU is evaluated in terms of confusion metrics as well, where it illustrates distinguished strengths over Cu-LSTMDNN, Cu-BLSTM, and Cu-GRU, as shown in Figure 7.

**Figure 8:**
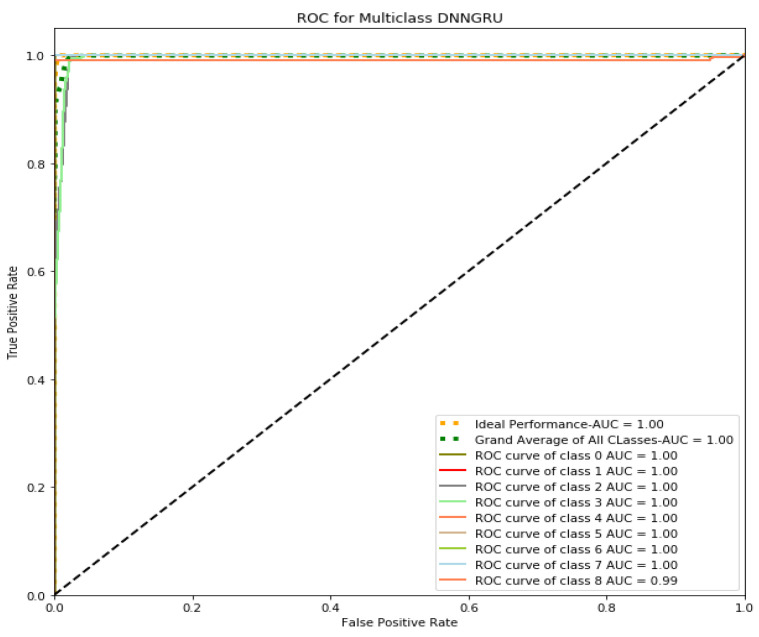
ROC curve analysis.

The operating performance of the DL classifiers can also be measured using the Receiver Operating Characteristics (ROC) Curve. An algorithm is used to determine the best possible threshold for a specific classification algorithm to increase the number of accurate results while minimizing false positives. TPR and FPR trade-offs can be determined by employing several measurements of probability thresholds, such as ROC Curves. The proposed Cu-DNNGRU is extensively evaluated along with Cu-LSTMDNN, Cu-BLSTM, and Cu-GRU. Figure 8 provides pictorial evidence regarding the superiority of the proposed framework.

**Figure 9:**
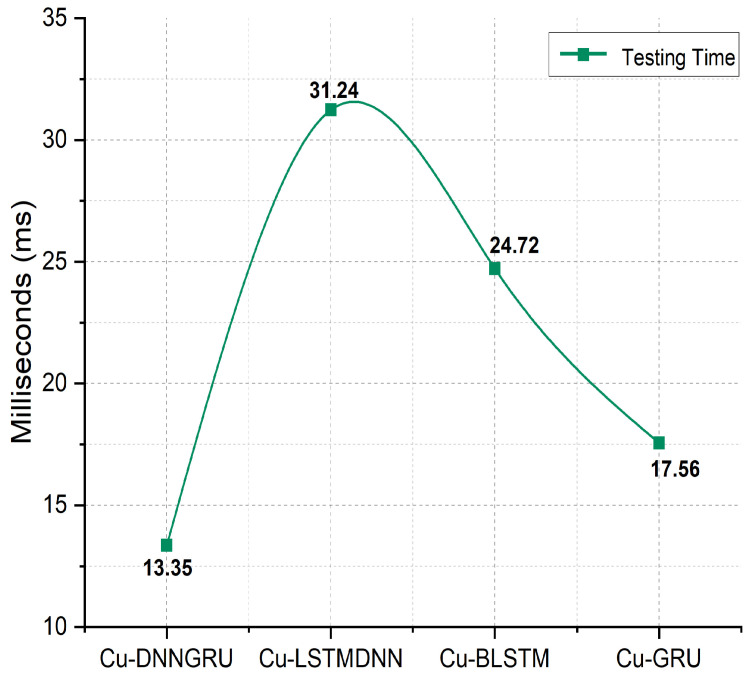
Testing time analysis.

Moreover, the training time is a crucial metric for assessing a system’s overall performance since it measures how long it takes for a plan to acquire the intrinsic sustainability of its absolute features. Figure 8 shows that the proposed Cu-DNNGRU has a training time of 13.35 ms, which is significantly less than the training time of Cu-LSTMDNN, Cu-BLSTM, and Cu-GRU, which consume the training times of 31.24 ms and 24.72 ms, and 17.6 ms, respectively, as pictorially elaborated in Figure 9.

The proposed Cu-DNNGRU is further compared with some state-of-the-art DL classifiers from the literature. The core objective of this extended performance evaluation is to obtain a comprehensive analytical idea regarding the performance of the proposed framework with its competitive algorithms. The performance comparison is conducted on the core performance parameters, i.e., accuracy, precision, recall, and F1-score. Table 7 summarizes this comparison where it can be transparently witnessed that the proposed Cu-DNNGRU has accomplished outstanding performance by outclassing some well-known benchmarked classifiers.

## 5. Conclusions

This research study is drafted about intrusion detection in RPL-based resource-constrained smart industries. We have proposed a Fog-assisted DL-enabled intrusion detection framework (Cu-DNNGRU) to interrogate a diversified array of potential security threats in smart industries. The under-contention model is trained on the N-BaIoT dataset, and its performance is evaluated on a reasonable spectrum of performance parameters equipped with accuracy, precision, recall, and F1-score. The proposed framework is then compared with several distinguished DL classifiers such as Cu-LSTMDNN, Cu-BLSTM, and Cu-GRU for comprehensive performance analysis. The performance is further investigated along with some benchmarked DL algorithms from the literature. The systematic simulation results validate the effectivity of the proposed model with 99.39% accuracy, 99.09% precision, 98.89% recall, and 99.21% F1-score. The designed framework has overwhelmed existing competitive schemes with dominant performance towards efficient intrusion detection against less consumption of system resources. Finally, we aim to train the proposed model on different datasets and enhance its detection strengths in the future. 

## Figures and Tables

**Table 1:** Existing literature.

Ref.	Year	Proposed Work	Classifier	Dataset	Limitations
[38]	2022	A security mechanism is devised for anomaly detection	ReLU PPO2	Oakridge Lab dataset	Computation overhead increases
[39]	2022	An analytical model is presented to filter organic traffic flows	CNN	CICDS2017, UNSW-NB15	Not suitable for resource-constrained environments
[40]	2022	A security framework is proposed for DOS detection	LSTMGRU, BLS	N-BaIoT	Communicational delays experienced
[41]	2022	An efficient IDS is designed for industrial IoT	LSTM	UNSW-NB15, NSL-KDD	Appropriate for small-scale networks only
[42]	2022	A malicious entity identification scheme is designed.	CNN	CICIDS2017	Demands significant system resources
[43]	2021	An extensive intrusion detection mechanism is constituted	DNN	NSL-KDD, UNSW-NB15	Extensive latencies have been noticed
[44]	2021	A multilayer threat classification model is proposed GRUCNN	ADFA-LD,	KDD99	Complexities increases in large-scale industrial networks
[45]	2021	Suspicious events detection scheme is designed	LSTM, SFLN	IoT-ID20	The communication stream is not stable
[46]	2021	A systematic approach is presented for abnormal traffic detection	PCA-DL	N-BaIoT	Extensive computational resources required
[47]	2021	A dynamic traffic analysis scheme is formulated	Binary, Multiclass	BOT-IoT	Highly resource consumptive
[48]	2020	An IDS is proposed for large-scale generic networks	DNN	Mirari dataset	Not efficient for medium-scale networks
[49]	2020	A secure communication framework is designed	Forest PS	BOT-IoT, CICIDS2017	Computational overhead increases
[50]	2020	The threat identification and classification model is designed	DT, MLP, LSTM	Customized dataset	Considerable increase in communication delays
[51]	2020	An extensive attacks analysis approach is proposed CART	CNN	NSL-KDD, KDD-99	Significantly complex for large-scale networks

**Table 2:** Proposed hybrid model details.

Algorithm	Layers	AF	Neurons	Optimizer	LF	Epochs	Batch-Size
Cu-DNNGRU	DNN Layer (4)	RELU	(400, 300, 200, 50)		CC-E		
	GRU LAYER (2)	RELU	(200, 100)		CC-E		
	Dropout	-	(0.7)	Adam		15	32
	Output Layer (1)	Softmax	(08)				
	Dense (2)	-	(200, 50)				
Cu-LSTMDNN	LSTM Layer (2)	RELU	(200, 100)		CC-E		
	DNN Layer (4)	RELU	(400, 300, 200, 50)		CC-E		
	Dropout	-	(0.7)	Adam		15	32
	Dense (2)	-	(200, 50)				
	Output Layer (1)	Softmax	(08)				
Cu-BLSTM	BLSTM Layer (4)	RELU	(400, 300, 200, 100)		CC-E		
	Output Layer (1)	Softmax	(08)				
	Dropout	-	(0.7)	Adam		15	32
	Dense (2)	-	(200, 50)				
Cu-GRU	GRU Layer (4)	RELU	(400, 300, 200, 100)		CC-E		
	Output Layer (1)	Softmax	(08)				
	Dropout	-	(0.7)	Adam		15	32
	Dense (2)	-	(200, 50)				

**Table 3:** N-BaIoT dataset details.

Attack	Attack Instances
Normal	61,400
Mirai Scan	4200
Mirai UDP	4161
Mirai Ack	4153
Mirai SYN	4200
Mirai UDP Plain	4165
Gafgyt Junk	4190
Gafgyt Combo	4220
Gafgyt TCP	4225
**Total**	**94,914**

**Table 4:** Experimental setup.

Processor	I7 (3.33 GHz)
OS Windows	10
RAM	16 GB
Language	Python
GPU	Geforce-1060
IDE	Spyder
Generation	8th
Libraries	Numpy, Tensorflow, Pandas, Keras, and Scikitlearn

**Table 5:** Class-wise detection accuracy.

Class	Cu-DNNGRU	Cu-LSTMDNN	Cu-BLSTM	Cu-GRU
Normal	99.92%	99.15%	98.96%	98.69%
Mirai Scan	99.86%	97.79%	97.29%	98.51%
Mirai UDP	98.89%	98.62%	97.61%	98.12%
Mirai Ack	99.71%	97.43%	98.10%	98.68%
Mirai SYN	99.68%	98.81%	97.62%	98.25%
Mirai UDP Plain	99.14%	98.89%	97.83%	97.89%
Gafgyt Junk	98.61%	96.61%	97.26%	98.64%
Gift Combo	99.85%	97.90%	98.15%	97.68%
Gafgyt TCP	99.12%	97.36%	97.25%	98.36%

**Table 6:** Cross-validation results.

Parameter	Models	F1	F2	F3	F4	F5	F6	F7	F8	F9	F10
Accuracy (%)	Cu-DNNGRU	98.61	98.89	99.14	99.12	99.14	99.86	99.68	99.71	99.85	99.92
	Cu-LSTMDNN	98.89	97.43	96.61	97.36	97.79	98.90	99.15	98.81	98.62	97.90
	Cu-BLSTM	98.96	98.15	97.62	97.26	97.61	97.25	97.31	97.83	97.29	98.10
	Cu-GRU	98.68	97.68	97.89	98.65	98.51	98.25	98.36	98.12	98.64	98.69
Recall (%)	Cu-DNNGRU	99.65	99.15	99.34	98.72	98.69	98.94	98.20	98.31	98.86	99.12
	Cu-LSTMDNN	99.15	98.69	98.45	98.94	98.78	98.65	98.15	98.14	97.56	97.37
	Cu-BLSTM	98.26	98.26	98.14	97.19	97.36	97.53	97.49	97.83	97.96	98.32
	Cu-GRU	98.52	98.34	98.91	98.79	98.86	99.35	98.65	98.61	98.69	98.54
F-score (%)	Cu-DNNGRU	98.99	99.41	99.34	99.11	99.11	98.89	99.14	99.29	99.24	99.81
	Cu-LSTMDNN	98.32	98.16	97.93	97.85	98.19	98.99	99.36	99.57	99.57	99.61
	Cu-BLSTM	97.65	97.86	97.95	97.81	97.73	97.49	97.72	99.19	98.64	97.53
	Cu-GRU	98.54	98.84	98.17	98.25	99.65	99.58	99.61	98.96	98.21	98.49
Precision (%)	Cu-DNNGRU	98.24	99.64	99.12	99.36	99.52	99.41	99.43	99.08	98.41	98.71
	Cu-LSTMDNN	98.65	97.56	97.35	97.56	97.51	97.46	97.1	97.25	97.36	98.42
	Cu-BLSTM	98.65	98.67	98.46	98.62	97.51	97.59	97.43	97.35	97.16	97.35
	Cu-GRU	99.15	98.46	97.69	97.54	98.53	98.15	97.56	98.36	98.13	98.69

**Table 7:** Comparison of the proposed model with recent threat detection approaches.

Ref.	Classifier	Dataset	Accuracy	Precision	Recall	F1-Score
Proposed	Cu-DNNGRU	N-BaIoT	99.39%	99.09%	98.89%	99.21%
[56]	BotStop	N-BaIoT	99.01%	99.02%	98.82%	98.09%
[57]	ELBA-IoT	N-BaIoT	98.79%	98.90%	98.59%	98.39%
[58]	XGB-RF	N-BaIoT	99.22%	98.99%	99.78%	99.09%
[59]	CNN	N-BaIoT	99.16%	98.97%	99.63%	99.04%

## Data Availability

Not applicable.
